# Genetic Analysis of *CYP1B1* and Other Anterior Segment Dysgenesis-Associated Genes in Latvian Cohort of Primary Congenital Glaucoma

**DOI:** 10.3390/biomedicines13051222

**Published:** 2025-05-18

**Authors:** Eva Elksne, Baiba Lace, Janis Stavusis, Anastasija Tvoronovica, Pawel Zayakin, Eriks Elksnis, Arturs Ozolins, Ieva Micule, Sandra Valeina, Inna Inashkina

**Affiliations:** 1 Department of Ophthalmology, Children’s Clinical University Hospital, Vienibas gatve 45, LV-1004 Riga, Latvia; 2 Department of Doctoral Studies, Riga Stradins University, Dzirciema 16, LV-1007 Riga, Latvia; 3 European Reference Network on Rare Eye Diseases (ERN-EYE), 67000 Strasbourg, France; 4 Latvian Biomedical Research and Study Centre, Ratsupites 1 k-1, LV-1067 Riga, Latviapawel@biomed.lu.lv (P.Z.); inna@biomed.lu.lv (I.I.); 5 Riga East Clinical University Hospital, Hipokrata 2, LV-1038 Riga, Latvia; 6 Latvian American Eye Centre, A.Deglava 12a, LV-1009 Riga, Latvia; 7 Department of Ophthalmology, Riga Stradins University, Dzirciema 16, LV-1007 Riga, Latvia; 8 Department of Surgery, Pauls Stradins Clinical University Hospital, Pilsonu 13, LV-1002 Riga, Latvia; 9 Faculty of Medicine, Riga Stradins University, Dzirciema 16, LV-1007 Riga, Latvia; 10 Department of Medical Genetics and Prenatal Diagnostics, Children’s University Hospital, Vienibas gatve 45, LV-1004 Riga, Latvia

**Keywords:** primary congenital glaucoma, *CYP1B1*, *FOXC1*, FOXE3, PXDN, PITX2, PITX3, PAX6, CPAMD8

## Abstract

**Background**: Primary congenital glaucoma (PCG) is a rare disease with an incidence of 1 in 12,000 to 18,000 in Europeans. The scarcity of the disease and limited access to genetic testing have hindered research, particularly within the Latvian population. **Objectives**: This study aims to present the preliminary results of a molecular genetic investigation into PCG in a Latvian cohort and to compare the prevalence of gene *CYP1B1* variants with other European studies as well as to the general population in Latvia. **Methods**: Twenty probands with clinically diagnosed PCG and 36 family members enrolled in the study. Genetic testing was conducted using genomic DNA from peripheral blood using next generation sequencing (NGS) of seven selected genes: *CYP1B1, FOXC1, FOXE3, PXDN, PITX2, PITX3, PAX6*, and *CPAMD8*. Four probands had whole-genome sequencing (WGS). **Results**: All participants were of European ancestry, with no family history of PCG. Most probands were diagnosed in their first year of life, with a female to male ratio of 1:1.2 and with 80.0% of cases being unilateral. No *CYP1B1* pathogenic variants were identified in the screened subjects. However, a heterozygous missense variant c.4357C>A (p.Pro4357Thr) in the *PXDN* gene was found in one proband and one of her parents that was classified as a variant of uncertain significance. **Conclusions**: This study represents the first genetic characterization of PCG in the Latvian population. Using NGS, we identified no pathogenic variants in the *CYP1B1* gene among affected individuals. Preliminary evidence from this cohort does not support *CYP1B1* variants as a predominant cause of PCG, though larger studies are needed to confirm this observation. Comprehensive genetic screening using whole-exome or whole-genome sequencing will be essential to identify the underlying genetic etiology of PCG in Latvia.

## 1. Introduction

Primary congenital glaucoma (PCG) is a rare disease worldwide with an estimated incidence of 1 in 12,000 to 18,000 in Europeans [1]. The previously reported incidence of PCG in Latvia was 5.3 cases per 100,000 live births, corresponding to 0.85 cases per 100,000 inhabitants between 2003 and 2017 [2]. The rarity of the disease and the lack of available genetic tests in recent decades have significantly impacted research in the field of PCG, especially in the Latvian population.

Given its severe clinical consequences, PCG is a potentially blinding disorder if left untreated. In most European cases, PCG develops in the first year of life and has a slight male predominance [1,2]. The Childhood Glaucoma Research Network (CGRN) defines PCG as glaucoma presenting without other congenital or acquired ocular/systemic conditions, typically accompanied by *buphthalmos* (ocular enlargement). PCG is subclassified by age of onset: neonatal (from birth till 1 month), infantile (above age of 1 month till 24 months), late-onset (above age of 24 months), or spontaneously arrested (*buphthalmos* with Haab’s striae, normal IOP, and healthy optic nerve) [3].

Among the genetic factors involved in PCG, pathogenic and likely pathogenic (P/LP) variants in the cytochrome P450 1B1 gene (*CYP1B1*, OMIM 601771) stand out as the most common and well-studied cause [4,5]. The gene encodes an enzyme, which is involved in the metabolism of various endogenous and exogenous compounds [6]. This enzyme plays a crucial role in the development and function of the trabecular meshwork and other ocular tissues responsible for the regulation of intraocular pressure (IOP) [7,8,9]. P/LP variants in *CYP1B1* disrupt these processes, leading to the pathophysiological changes observed in PCG like increased IOP, optic nerve excavation, Haab’s striae, and corneal and scleral enlargement [10,11].

Studies have identified a wide spectrum of *CYP1B1* pathogenic variants associated with PCG, with variations in the presentation and severity of the disease often linked to specific genetic changes [12,13,14]. The incidence of *CYP1B1* pathogenic variants varies between different populations, reflecting genetic diversity and possible influence of modifying genes and environmental factors [15,16]. Understanding the role of *CYP1B1* variants in the development of PCG is essential to develop targeted genetic tests, early diagnosis, and personalized treatment strategies [17].

As PCG represents a significant health concern in Latvia, with prevalence rates mirroring broader European trends, there is a pressing need for population-specific research. Our study addresses this gap, while supporting the Latvian healthcare system priorities of enhanced pediatric screening, advanced treatments, and genetic counselling [2]. The purpose of the study is to describe the primary results of molecular genetic testing in a cohort of PCG and to compare the prevalence of *CYP1B1* pathogenic variants with other PCG studies in Europe as well as to the general population in Latvia.

## 2. Materials and Methods

### 2.1. Subjects

Between January 2003 and December 2021, 29 consecutive patients with PCG were treated at a tertiary medical centre and included in the Latvian registry of PCG. The registry involves all clinically validated cases in the country since Children’s Clinical University Hospital is the only place in Latvia where PCG is diagnosed. Of all registered cases, 9 were excluded (5 declined participation, 4 were lost to follow-up), leaving 20 probands and 36 family members enrolled in the study. The study cohort comprised 20 probands (11 male (55.0%), 9 female (45.0%)), with a female-to-male ratio of 1:1.2. The median age at diagnosis was 7.0 months (IQR 3.75; range: 2.0–18.0 months). The diagnostic criteria of PCG were at least 2 criteria of the following according to CGRN [3]:

- IOP above 21 mmHg;
- Haab striae, increased corneal diameter (>11 mm in newborns, >12 mm in children younger than 1 year old, and >13 mm in children older than 1 year old);
- Glaucomatous changes in the optic nerve disc (cup–disc asymmetry of >0.2, focal rim thinning, or progressive increase in cup–disc ratio);
- Increased ocular dimensions that outpace normal growth (*buphthalmos*).

Consanguinity was systematically assessed through standardized patient interviews documenting familial relationships across three generations.

All other secondary causes of congenital or acquired glaucoma were excluded.

Individuals with PCG have been enrolled in the Genome Database of the Latvian Population as part of the European Regional Development Fund (ERDF) research project “The determination of the causative mechanisms of rare inherited diseases using the whole genome sequencing approach”. All matters related to consent and data handling for the genetic research of the participants are governed by the approvals of the Latvian Central Committee of Medical Ethics (Protocol No.01-29.1.2./6407) and the Ethics Committee of Riga Stradins University (protocol No 2-PĒK-4/46/2022).

The frequencies of *CYP1B1* variants in the Latvian general population were obtained from a study conducted as part of project 4.1.1.r.0/3/22/I/VM/001, “Establishment of the Latvian Population Genome Reference”, which involved whole-genome sequencing (WGS) of 605 individuals from Latvia. The study design is described elsewhere [18].

All procedures were conducted in accordance with the principles of the Declaration of Helsinki. Written informed consent was obtained from participants above 18 years of age or their parents/guardians if younger than 18 years.

### 2.2. Massive Parallel Sequencing

Total DNA was extracted from peripheral blood leukocytes using the standard phenol/chloroform method. DNA concentration was determined using NanoDrop^®^ ND-1000 (NanoDrop Inc., Wilmington, DE, USA).

For gene-panel next-generation sequencing (NGS), 500 ng of high-quality genomic DNA was mechanically fragmented. Libraries were prepared from the fragmented DNA using the Twist Library Preparation Kit with Amp Mix, Mechanical Fragmentation (Twist Bioscience, South San Francisco, CA, USA), custom-synthesized Equinox Library Amp Mix, and all necessary TWIST-compatible reagents, following the manufacturer’s instructions. The obtained libraries were converted using the MGIEasy Library Conversion kit (MGI Tech Co., Shenzhen, China). The libraries were sequenced at a mean coverage of 1500–3000 X on the MGI platform DNBSEQ-G4000, using the nanoball approach and paired-end 150 bp reads. *CYP1B1*, *FOXC1*, *FOXE3*, *PXDN*, *PITX2*, *PITX3*, *PAX6*, and *CPAMD8* genes were analyzed. The gene panel encompassed all anterior segment dysgenesis-related genes (ASGD1–ASGD8, OMIM 107250, 610256, 601631, 137600, 604229, 617315, 269400, 617319), prioritizing the following: established PCG-associated genes (*CYP1B1*, *FOXC1*, *PXDN*, *PITX2*, *CPAMD8*) and genes with potential phenotypic overlap (*FOXE3*, *PITX3*, *PAX6*).

For WGS, libraries were prepared from 300 ng of high-quality genomic DNA using the MGIEasy Universal DNA Library Prep Set (MGI Tech Co., Shenzhen, China) following the manufacturer’s instructions. The libraries were sequenced at a mean coverage of 16–35 X on the MGI platform MGISEQ-2000RS, using the nanoball approach and paired-end 150 bp reads.

### 2.3. Bioinformatics Analyses of NGS Data and Variant Filtering

Output FASTQ files were processed, including the trimming of low-quality read ends (QC < 20) and adaptor sequences using Cutadapt software 4.7 [19], followed by quality control of the obtained sequencing reads with FastQC. FastQC generated per-base sequence quality score plots, duplication rate charts, and a range of other key quality control metrics.

Samples meeting quality standards were then aligned to the human reference genome (GRCh38) using Bowtie2 software 2.5.4 [20]. Subsequently, local realignment around indels, base quality score recalibration, and variant calling were performed using BCFtools [21]. The sequencing coverage for each tested sample was calculated based on the alignments. 

For samples prepared using the Twist amplicon procedure, off-target reads were filtered out, retaining only those matching the amplicon regions. For WGS analysis, a customized bioinformatics pipeline was developed, utilizing BCFtools 1.21 [21] as the core computational tool, integrated into an ad hoc workflow in the R programming language. The workflow includes the addition of 1000 Genomes annotations and sex checks, as well as analysis using ExpansionHunter v5.0.0 [22], MANTA 1.6.0 [23], and AnnotSV 2.2 [24].

Finally, the obtained results were annotated using VEP 99 [25] and uploaded to the SEQR 0.3.0 [26] database for user-friendly analysis. Variant filtration focused on rare variants in coding regions and splice sites predicted to affect protein function and following the known inheritance pattern for the disease-associated genes. Variants were functionally annotated based on data from SiFT, CADD, GnomAD, and Polyphen 2.

All detected gene variants were classified according to the guidelines of the American College of Medical Genetics and Genomics and the Association for Molecular Pathology (ACMG/AMP) [27], updated by the ClinGen Sequence Variant Interpretation Working Group (2020). For variant interpretation, we used established databases including ClinVar and gnomAD and the variant annotation and interpretation platform MobiDetails [28] to support pathogenicity assessment and ensure consistency with current clinical and research standards.

### 2.4. PCR and Sanger Sequencing

Reported variants were validated by direct sequencing using 10 pm of each primer (primer sequences are available upon reasonable request), the BigDye™ Terminator v3.1 Cycle Sequencing Kit (ThermoFisher Scientific, Waltham, MA, USA), and an ABI PRISM 3100 sequencer (Applied Biosystems, Foster City, CA, USA).

### 2.5. Statistical Data Analysis

The median and interquartile range (IQR) were used to describe the age at diagnosis (in months) in PCG cases.

To compare the observed frequency of the *CYP1B1* variant in our cohort with the gnomAD reference European non-Finnish population (v4.1.0.), we performed a two-sided Fisher’s exact test. Contingency tables were constructed to compare variant counts between the two groups, and statistical significance was assessed using an exact test. Benjamini–Hochberg false discovery rate correction with α = 0.05 was applied to account for multiple comparisons and only reported when statistically significant. The data were analyzed using statistical software (IBM Corp. Released 2016. IBM SPSS Statistics for Macintosh, Version 24.0; Armonk, NY, USA) with significance defined as *p* < 0.05.

## 3. Results

Our study enrolled 20 probands and 36 family members. All families were unrelated and were permanently living in Latvia. All patients and their parents were European decent. Based on parental reports, the ethnic distribution was as follows: 11 Latvian, 6 Russian, and 1 Ukrainian family. Two families chose not to disclose their ethnicity. No family members were affected by PCG, and no positive family history or consanguinity were reported. Additionally, the BI pipeline for gene panel sequencing analysis included relatedness parameter analysis. The results consistently showed scores characteristic of non-consanguineous marriages, confirming the absence of significant familial relationships among the studied individuals.

Table 1 describes the profiles of patients with PCG included in the study. The median age at diagnosis was 7.0 months (IQR 3.75) and 75.0% of cases were diagnosed before 9 months of age. Between 2003 and 2021, only cases with infantile onset (age of onset from 1 month till 24 months) PCG were diagnosed and included in the study. Most probands were treated in the first year of life. The majority of cases were unilateral (80.0%). The diagnosis of unilateral or bilateral PCG cases was established clinically following CGRN classification [3].

In this study, we identified no pathogenic *CYP1B1* variants associated with PCG among the screened subjects (both probands and family members). In addition to *CYP1B1* analysis, all participants underwent primary genetic testing using a panel of genes associated with anterior segment dysgenesis, including *FOXC1, FOXE3, PXDN, PITX2, PITX3, PAX6*, and *CPAMD8*.

Genetic testing revealed that proband PCG-11 carried a heterozygous missense variant in the *PXDN* gene (c.4357C>A, p.Pro1453Thr, rs1172607325), resulting in a proline-to-threonine substitution. The same variant was identified in one unaffected parent. According to ClinVar, this variant is classified as being of uncertain significance, with PM1—known mutational hot-spot and/or well-established functional domain and PM2—extremely low frequency in gnomAD database (0.00001020 in European non-Finnish population and not found in the Latvian Population Genome Reference Database) [29]. In silico prediction tools showed controversial results, with CADD score being 21.9, conservation score Phylop 5.37, and no effect on splicing by SpliceAI 0.00.

No pathogenic variants were detected in the remaining genes (*FOXC1, FOXE3, PITX2, PITX3, PAX6* or *CPAMD8*) for either probands or their relatives.

Table 2 summarizes the protein-coding *CYP1B1* variants identified in our cohort of 20 patients. The observed allele frequencies showed close alignment with both the Latvian reference population and European (non-Finnish) genetic ancestry groups according to the gnomAD database. Statistical analysis revealed no significant differences in variant frequencies between our study population and these reference groups (all *p* > 0.05). In addition, analysis of the Latvian Population Genome Reference Database revealed no carriers of pathogenic *CYP1B1* variants in the general Latvian population.

WGS of the four patients failed to identify pathogenic variants in genes associated with primary/secondary congenital glaucoma or anterior segment dysgenesis. Genes screened in the analysis comprised *LTBP2, TEK, MYOC, WDR36, NTF4, SH3PXD2B, LMX1B, FBN1,* and *NF1*, along with other relevant candidates (in total 363 genes related to glaucoma were tested) [17,30,31].

## 4. Discussion

We presented the first results of genetic testing of 20 PCG patients and 36 of their first-degree family members in Latvia. The aim of this study was to investigate the frequency of *CYP1B1* pathogenic variants in a cohort of 20 unrelated Latvian families with PCG. Our results revealed no pathogenic variants in the *CYP1B1* gene applying the NGS methods.

Our results could be explained by several aspects. The Latvian cohort of PCG included only sporadic cases (defined as those without affected relatives in at least three generations of family history). There were no familial cases reported, as no family member was diagnosed with PCG. According to the literature, *CYP1B1* pathogenic variants may have a high prevalence in familial cases, up to 80–100% [32,33]. A previously high prevalence of *CYP1B1* pathogenic variants has been described in Slovak Roma, representing 100% of the cases tested [32]. Such a high prevalence of *CYP1B1* pathogenic variants has been associated with consanguinity, which was significant in the described Slovak Roma populations. Our cohort did not include any cases of consanguineous marriage, which is not typical for Latvian and, in general, for other European populations. Last, but not least, the number of cases tested was only 20, although the study included most of all cases diagnosed and treated between 2003 and 2021. A previous study revealed that the incidence of PCG in Latvia is about 5.3 cases in 100,000 live born children, accounting for about 1–2 new cases per year in such a small population [2]. Compared to other small studies, like those in Switzerland (14 probands), Russia (25 probands), and France (31 probands), a part of the cases were caused by *CYP1B1* pathogenic variants; however, these studies included relatively few probands [34,35,36].

Our failure to identify pathogenic *CYP1B1* variants in this comprehensive national cohort may suggest several population-specific factors that warrant careful consideration:

(1) Population-specific genetic architecture: The distinct Baltic genetic background may harbour alternative PCG-associated loci or protective variants that modify *CYP1B1* expression.
(2) Modifier gene effects: Potential interactions with other glaucoma-related genes (e.g., *TEK, LTBP2*) cannot be excluded.
(3) Technical considerations: While our comprehensive sequencing covered all coding regions, deep intronic or regulatory variants would require WGS for detection.

These findings may underscore the importance of expanding genetic screening to non-*CYP1B1* targets in Baltic populations, establishing regional mutation databases for precision diagnosis and investigating potential environmental modifiers in low-prevalence groups.

### 4.1. CYP1B1 Gene Function and Pathogenic Mechanisms in PCG

The *CYP1B1* gene located in 2p22.2 consists of three exons (only exon 2 and exon 3 are coding) and two introns and encodes a protein of 543 amino acids [14]. The *CYP1B1* gene produces monomeric mixed-function monooxygenase, which belongs to the cytochrome P450 superfamily [37]. *CYP1B1* is strongly expressed in the anterior segment of the eye (in the iris, trabecular meshwork, ciliary body), playing a role in secreting aqueous humour and regulating outflow facility [38]. The exact mode of *CYP1B1* involvement in the pathogenesis of PCG is still uncertain, though several theories have been raised, such as dysfunctional retinoid acid metabolism and defectiveness in the 17β-estradiol pathway reducing coded enzyme activity or protein stability and function, as well as retinal ganglion cells axonopathy caused by *CYP1B1* pathogenic variants [15,17,39,40,41]. A study on homozygous *CYP1B1* gene knockout mice demonstrated malformations in ocular drainage structures, confirming the role of the gene in the development of ocular abnormalities [42]. Doshi et al. indicated that the developmental abnormalities in the trabecular meshwork associated with PCG may stem from inadequate or absent metabolism of essential endogenous substrates in the ciliary epithelium, resulting from a non-functional CYP1B1 enzyme [10]. Williams et al. demonstrated that CYP1B1 plays a critical role in ocular fissure closure by modulating neural crest cell migration and differentiation through retinoic acid independent mechanisms. Their findings suggest that targeted, ocular-specific upregulation of *CYP1B1* expression may represent a promising therapeutic strategy for PCG [43].

Pathogenic variants in *CYP1B1* have been known to cause not only PCG but also anterior segment dysgenesis 6 (e.g., Peters anomaly, Axenfeld–Rieger anomaly, *ectropion uveae* with partial aniridia and complete aniridia) [44,45,46,47,48] and juvenile open-angle glaucoma (JOAG), and can even significantly increase the risk for primary open-angle glaucoma (POAG) [17,38,49,50]. López-Garrido et al. hypothesized that PCG, JOAG, and POAG may all arise from varying degrees of goniodysgenesis caused by differing levels of CYP1B1 activity, along with modifications from specific genes or environmental factors [15].

### 4.2. CYP1B1 Variants in Other Populations

The prevalence of *CYP1B1* pathogenic variants in PCG cases varies considerably across different European populations as described in Table 3. The highest prevalence has been reported in the Roma population in Slovakia (100%), followed by screened probands in Portugal (73.3%), France (48.4%), and Spain (34.8%) [15,32,34,51]. The mean prevalence in described European populations is around 40% of cases caused by *CYP1B1* pathogenic variants (Table 3).

Previous studies have described that *CYP1B1* pathogenic variants are the main cause of familial cases and above 20% of sporadic cases [52]. Furthermore, while a substantial percentage of *CYP1B1* pathogenic variants have been identified in Europeans, a considerable number of individuals with PCG still have unknown genetic causes [17]. A systematic review by Kumar et al. demonstrated that *CYP1B1* variants exhibit significant geographic and population-specific variability in primary congenital glaucoma, with reported prevalence rates ranging from 5% to 86% worldwide. The most frequently observed pathogenic variants included p.Gly61Glu (G61E), p.Arg368His (R368H), p.Glu229Lys (E229K), and p.Arg390His (R390H) [53].

**Table 3:** Prevalence of *CYP1B1* pathogenic variants in European populations. Most cases were of European decent; however, several cases of different origin were reported. (N/A not applicable). * Identified 11 different mutations. Four patients were compound heterozygotes, two subjects heterozygous, and one homozygous. ** Probands with 1 or 2 sequence variations in *CYP1B1*.

Country	Probands	Number of Patients with Pathogenic Variants in *CYP1B1* (%)	Most Frequent Variant
Spain [15]	161	56 (34.8%)	p.E387K (rs55989760)
Italy [54]	72	25 (34.7%)	p.G61E (rs289367000)
Germany [55]	39	7 (18.0%)	N/A *
France [34]	31	15 (48.4%)	g.4340delG
Denmark [56]	37	10 (27.0%) **	p.W57 * (rs72549387)
Portugal [51]	30	22 (73.3%)	p.A179fs (rs771076928)
Russia [35]	25	5 (25.0%)	p.R444Q (rs72549376) p.R444 * (rs377049098)
Slovakia (Roma population) [32]	20	20 (100%)	p.E387K (rs55989760)
Switzerland [36]	14	6 (42.9%)	p. L487P (rs1682415237) p.R355fs (rs72549380) c.1044-3C>T (rs761216127)
Latvia (this study)	20	0	N/A

*CYP1B1* variants are the most common cause of PCG worldwide. Outside Europe, the highest prevalence rates of *CYP1B1* alterations have been registered in Saudi Arabia (80–100%) and Iran (70%) [4,33]. Furthermore, significantly lower prevalence has been described in Japan (20%) and the Chinese Han population (17.2%) [4]. A meta-analysis has described founder pathogenic variants that are more frequent in different PCG populations—for example, g.4339delG in Morocco, p.G61E in Saudi Arabia, Iran, and Lebanon, p.R390H in Pakistan, and p.E387K in Europe [57]. Pathogenic variant p.E387K has been described in Spain and the Roma population in Slovakia [15,32]. The Slovak Roma population was the first population identified with PCG resulting from a single variant in the *CYP1B1* gene and about 10% of healthy Roma were found to carry this allele [32].

The population of Latvia contains a relatively high proportion of ancestry from two major early European groups: West European hunter-gatherers and the Yamnaya. The genetic diversity found in the population is similar to that of other European populations [58,59,60]. The first WGS study in Latvia identified over 18.2 million genetic variants, of which 3.3% were not yet represented in the gnomAD and dbSNP databases. Furthermore, the researchers observed that the Latvian cohort forms a notable and distinct cluster within European subpopulations [18]. The genetic difference from other well-described populations with PCG, such as in Saudi Arabia, India, and the Roma in Slovakia, could be one of the reasons why other pathogenic variants in different genes may be population-specific in Latvia [58]. Unfortunately, there are no studies available regarding PCG genetics from neighbouring Baltic countries that could be more closely related to Latvia.

### 4.3. Genetic Landscape of PCG Beyond CYP1B1

Today, five gene loci have been described for PCG–GLC3A (gene *CYP1B1*), GLC3B (corresponding gene unknown), GLC3C (corresponding gene unknown), GLC3D (gene *LTBP2*), and GLC3E (gene *TEK*). There are also several candidate genes (*MYOC, FOXC1, FOXC2, PITX2*) and other genes reported (*GUCA1C, WDR36, OPA1, NTF4, COL1A1, PXDN, SH3PXD2B, CPAMD8, TNF-α*) that may play a minor role in the development of PCG. *CYP1B1* is defined as a core gene of PCG together with the *LTBP2, MYOC,* and *TEK* genes [17].

Genetic testing for this study, in addition to *CYP1B1* sequencing, included the *FOXC1, FOXE3, PXDN, PITX2, PITX3, PAX6*, and *CPAMD8* genes. Table 4 provides a summary of the other genes tested in this study.

Pathogenic variants in the *PXDN* gene (peroxidasine, OMIN 269400, located in 2p25.3) are responsible for anterior segment dysgenesis 7 (ASGD7) in the patterns of autosomal recessive inheritance [67]. The proband PCG-11 had a heterozygous missense variant *PXDN* (NM 012293.3) c.4357C>A (p.Pro4357Thr) in one allele, and the same changes were found in one of her parents. According to ClinVar and VarSome databases, this variant is classified as ‘uncertain significance’ [29,81]. No pathogenic variants in the genes *FOXC1, PITX2,* and *CPAMD8* were found during this study, although these have been identified in a few cases of PCG [16,17,70,79].

Testing for *FOXC1* could be helpful to prevent misdiagnosis and to exclude cases related to secondary congenital glaucoma as the clinical features of Axenfeld–Rieger anomaly/syndrome could be subtle [17,82]. Possibly, the level of residual *FOXC1* activity may potentially determine the development of PCG rather than Axenfeld–Rieger syndrome/anomaly and pathogenic variants could be associated with PCG, too [17,36,61]. In addition, *PITX2* has been described as a modifier gene for PCG [16].

### 4.4. Limitations of the Study

There are several limitations of the study that we would like to address.

First of all, the small number of cases tested is noteworthy. The rarity of the disease in Latvia has a significant impact on the genetic screening of probands, while not all cases are registered (before 2003) and not all known patients agreed to participate in the study. The official registry only includes cases from 2003 onwards. Several factors limited recruitment of pre-2003 cases: (1) unavailability of patient contact information; (2) lack of response from local ophthalmologists regarding previous cases; and (3) non-participation of some eligible patients. We acknowledge that the limited cohort size may have constrained our ability to detect certain genetic associations. Expanding this research through both broader national sampling and collaborative Baltic studies would significantly advance our understanding of PCG etiology in our region.

Second, our cohort had no cases of familial PCG and neonatal onset, which are more likely to be explained by *CYP1B1* pathogenic variants.

Third, pathogenic variants could have been missed because of the screening technique used. While our comprehensive sequencing covered all coding regions, deep intronic or regulatory variants would require WGS for detection. Previous studies have demonstrated that reduced *CYP1B1* promoter activity in risk alleles plays a crucial role in PCG pathogenesis [83]. The gene panel did not cover intronic regions, whereas WGS encompassed the entire genome, enabling analysis of deep intronic variants. Promoter regions were only partially analyzed.

## 5. Conclusions

This study represents the first genetic characterization of PCG in the Latvian population. Using NGS, we identified no pathogenic variants in the *CYP1B1* gene among affected individuals. Preliminary evidence from this cohort does not support *CYP1B1* variants as a predominant cause of PCG, though larger studies are needed to confirm this observation. Comprehensive genetic screening using whole-exome or whole-genome sequencing will be essential to identify the underlying genetic etiology of PCG in Latvia.

## Figures and Tables

**Table 1:** Patient profiles with PCG included in the study (F—female; M—male; OD—right eye; OS—left eye; IOP—intraocular pressure; WGS—whole-genome sequencing).

	Sex	Year of Birth	Ethnicity	Cosanguinity	Age at Diagnosis (Months)	Affected Eye	IOP at Diagnosis (Affected Eye or Both (OD/OS)), (mmHg)	Genetic Testing
PCG-1	M	2002	Russian	No	4	OD	27	Gene-panel
PCG-2	M	2003	Latvian	No	7	OS	30	Gene-panel
PCG-3	F	2004	Russian	No	8	OD	27	Gene-panel; WGS
PCG-4	F	2004	Latvian	No	4	OD/OS	22/24	Gene-panel; WGS
PCG-5	M	2006	Latvian	No	4	OD	27	Gene-panel
PCG-6	F	2008	Russian	No	5	OD/OS	25/26	Gene-panel; WGS
PCG-7	M	2008	Latvian	No	10	OD/OS	22/22	Gene-panel
PCG-8	F	2009	Russian	No	18	OD	24	Gene-panel
PCG-9	F	2011	Latvian	No	10	OD/OS	22/22	Gene-panel
PCG-10	M	2011	Russian	No	5	OS	31	Gene-panel
PCG-11	F	2012	Latvian	No	2	OD	22	Gene-panel
PCG-12	M	2014	Latvian	No	8	OS	44	Gene-panel
PCG-13	M	2015	Latvian	No	6	OD	30	Gene-panel
PCG-14	F	2015	Latvian	No	7	OD	27	Gene-panel
PCG-15	M	2015	Russian	No	7	OS	23	Gene-panel, WGS
PCG-16	M	2017	Not reported	No	8	OS	23	Gene-panel
PCG-17	F	2018	Latvian	No	8	OS	42	Gene-panel
PCG-18	F	2019	Latvian	No	7	OS	31	Gene-panel
PCG-19	M	2020	Ukrainian	No	9	OD	25	Gene-panel
PCG-20	M	2020	Not reported	No	18	OD	26	Gene-panel

**Table 2:** Protein-coding *CYP1B1* variants identified in 20 patients in this study. * Frequency of CYP1B1 variant in this study and Latvian population. ** Frequency of CYP1B1 variant in this study and gnomAD (European non-Finnish population).

Variant	Rs Code	Incidence in This Study (Alleles)	Frequency in This Study	Frequency in Latvia	*p* Value *	Gnomad Frequency	*p*-Value **	SIFT	Poly Phen-2	CADD	Clinvar Classification
c.1358A>G, p.Asn453Ser	rs1800440	7	0.1750	0.1360	0.48	0.1864	0.85	No prediction	No prediction	23.70	Benign/Likely benign
c.1294C>G, p.Leu432Val	rs1056836	19	0.4750	0.3890	0.27	0.4409	0.66	No prediction	No prediction	8.95	Benign
c.142C>G p.Arg48Gly	rs10012	15	0.3750	0.3890	0.86	0.2813	0.19	No prediction	No prediction	9.70	Benign/Likely benign
c.355G>T p.Ala119Ser	rs1056827	15	0.3750	0.4070	0.69	0.2852	0.21	No prediction	No prediction	4.73	Benign/Likely benign
c.1347T>C p.Asp449=	rs1056837	25	0.6250	0.6110	0.86	0.5597	0.41	No prediction	No prediction	0.66	Benign

**Table 4:** Description of genes *FOXC1, FOXE3, PXDN, PITX2, PITX3, PAX6*, and *CPAMD8* (AD—autosomal dominant; AR—autosomal recessive; PCG—primary congenital glaucoma; ASGD—anterior segment dysgenesis). * Few cases have been reported.

**Gene**	**OMIN**	**Locus**	**Mode of Inheritance**	**Phenotypes**	**Associated with PCG**	**References**
*FOXC1*	601090	6p25.3	AD	ASGD 3 (Peters anomaly, Axenfeld anomaly, Rieger anomaly, iris hypoplasia, aniridia), Axenfeld–Rieger syndrome, PCG	Yes	[17,36,61,62,63,64]
*FOXE3*	601094	1p33	AR or AD	ASGD 2 (congenital primary aphakia, cataract, Peters anomaly, microphthalmia)	No	[65,66]
*PXDN*	269400	2p25.3	AR	ASGD 7 (Corneal opacity, congenital cataract, microcornea)	Yes *	[17,67,68,69,70]
*PITX2*	601542	4q25	AD	Axenfeld–Rieger syndrome, ASGD 4 (Peters anomaly, iris hypoplasia), ring dermoid of cornea, PCG	Yes	[16,17,71,72,73]
*PITX3*	602669	10q25	AD or AR	ASGD 1, Congenital posterior polar cataract	No	[74,75]
*PAX6*	607108	11p13	AD	Anaridia, ASGD 5 (Peters anomaly, Axenfeld anomaly, and Rieger anomaly) keratitis, foveal hypoplasia, congenital cataract, optic nerve hypoplasia	No	[76,77,78]
*CPAMD8*	608841	19p13.11	AR	ASGD 8 (iris hypoplasia, ectopia lentis, corectopia, ectropion uveae, cataract)	Yes *	[79,80]

## Data Availability

The original contributions presented in this study are included in the article. Further inquiries can be directed to the corresponding author.

## References

[1] Spaeth G.L. (2021). European Glaucoma Society Terminology and Guidelines for Glaucoma, 5th Edition. Br. J. Ophthalmol..

[2] Elksne E., Baumane K., Ozolins A., Valeina S. (2021). The epidemiological and clinical findings from the latvian registry of primary congenital glaucoma and evaluation of prognostic factors. Medicina.

[3] Thau A., Lloyd M., Freedman S., Beck A., Grajewski A., Levin A.V. (2018). New classification system for pediatric glaucoma: Implications for clinical care and a research registry. Curr. Opin. Ophthalmol..

[4] Shah M., Bouhenni R., Benmerzouga I. (2022). Geographical Variability in CYP1B1 Mutations in Primary Congenital Glaucoma. J. Clin. Med..

[5] Yu-Wai-Man C., Arno G., Brookes J., Garcia-Feijoo J., Khaw P.T., Moosajee M. (2018). Primary congenital glaucoma including next-generation sequencing-based approaches: Clinical utility gene card. Eur. J. Hum. Genet..

[6] Nebert D.W., Russell D.W. (2002). Clinical importance of the cytochromes P450. Lancet.

[7] Bejjani B.A., Xu L., Armstrong D., Lupski J.R., Reneker L.W. (2002). Expression patterns of cytochrome P4501B1 (Cyp1b1) in FVB/N mouse eyes. Exp. Eye Res..

[8] Zhao Y., Sorenson C.M., Sheibani N. (2015). Cytochrome P450 1B1 and Primary Congenital Glaucoma. J. Ophthalmic Vis. Res..

[9] Zhao Y., Wang S., Sorenson C.M., Teixeira L., Dubielzig R.R., Peters D.M., Conway S.J., Jefcoate C.R., Sheibani N. (2013). Cyp1b1 mediates periostin regulation of trabecular meshwork development by suppression of oxidative stress. Mol. Cell Biol..

[10] Doshi M., Marcus C., Bejjani B.A., Edward D.P. (2006). Immunolocalization of CYP1B1 in normal, human, fetal and adult eyes. Exp. Eye Res..

[11] Hollander D.A., Sarfarazi M., Stoilov I., Wood I.S., Fredrick D.R., Alvarado J.A. (2006). Genotype and phenotype correlations in congenital glaucoma: CYP1B1 mutations, goniodysgenesis, and clinical characteristics. Am. J. Ophthalmol..

[12] Genčík A., Genčíkova A., Ferák V. (1982). Population genetical aspects of primary congenital glaucoma. I. Incidence, prevalence, gene frequency, and age of onset. Hum. Genet..

[13] Abu-Amero K.K., Osman E.A., Mousa A., Wheeler J., Whigham B., Allingham R.R., Hauser M.A., Al-Obeidan S.A. (2011). Screening of CYP1B1 and LTBP2 genes in Saudi families with primary congenital glaucoma: Genotype-phenotype correlation. Mol. Vis..

[14] Bejjani B.A., Lewis R.A., Tomey K.F., Anderson K.L., Dueker D.K., Jabak M., Astle W.F., Otterud B., Leppert M., Lupski J.R. (1998). Mutations in CYP1B1, the gene for cytochrome P4501B1, are the predominant cause of primary congenital glaucoma in Saudi Arabia. Am. J. Hum. Genet..

[15] López-Garrido M.P., Medina-Trillo C., Morales-Fernandez L., Garcia-Feijoo J., Martínez-de-la-Casa J.M., García-Antón M., Escribano J. (2013). Null CYP1B1 genotypes in primary congenital and nondominant juvenile glaucoma. Ophthalmology.

[16] Medina-Trillo C., Aroca-Aguilar J.D., Ferre-Fernández J.J., Alexandre-Moreno S., Morales L., Méndez-Hernández C.D., García-Feijoo J., Escribano J. (2019). Role of FOXC2 and PITX2 rare variants associated with mild functional alterations as modifier factors in congenital glaucoma. PLoS ONE.

[17] Leysen L., Cassiman C., Vermeer S., Casteels I., Balikova I. (2022). Genetics in primary congenital glaucoma: Implications in disease management and counseling. Eur. J. Med. Genet..

[18] Reščenko R., Brīvība M., Atava I., Rovīte V., Pečulis R., Silamiķelis I., Ansone L., Megnis K., Birzniece L., Leja M. (2023). Whole-Genome Sequencing of 502 Individuals from Latvia: The First Step towards a Population-Specific Reference of Genetic Variation. Int. J. Mol. Sci..

[19] Martin M. (2011). Cutadapt removes adapter sequences from high-throughput sequencing reads. EMBnet J..

[20] Langmead B., Salzberg S.L. (2012). Fast gapped-read alignment with Bowtie 2. Nat. Methods.

[21] Danecek P., Bonfield J.K., Liddle J., Marshall J., Ohan V., Pollard M.O., Whitwham A., Keane T., McCarthy S.A., Davies R.M. (2021). Twelve years of SAMtools and BCFtools. Gigascience.

[22] Dolzhenko E., Deshpande V., Schlesinger F., Krusche P., Petrovski R., Chen S., Emig-Agius D., Gross A., Narzisi G., Bowman B. (2019). ExpansionHunter: A sequence-graph-based tool to analyze variation in short tandem repeat regions. Bioinformatics.

[23] Chen X., Schulz-Trieglaff O., Shaw R., Barnes B., Schlesinger F., Källberg M., Cox A.J., Kruglyak S., Saunders C.T. (2016). Manta: Rapid detection of structural variants and indels for germline and cancer sequencing applications. Bioinformatics.

[24] Geoffroy V., Herenger Y., Kress A., Stoetzel C., Piton A., Dollfus H., Muller J. (2018). AnnotSV: An integrated tool for structural variations annotation. Bioinformatics.

[25] McLaren W., Gil L., Hunt S.E., Riat H.S., Ritchie G.R., Thormann A., Flicek P., Cunningham F. (2016). The Ensembl Variant Effect Predictor. Genome Biol..

[26] Pais L.S., Snow H., Weisburd B., Zhang S., Baxter S.M., DiTroia S., O’Heir E., England E., Chao K.R., Lemire G. (2022). seqr: A web-based analysis and collaboration tool for rare disease genomics. Hum. Mutat..

[27] Richards S., Aziz N., Bale S., Bick D., Das S., Gastier-Foster J., Grody W.W., Hegde M., Lyon E., Spector E. (2015). Standards and guidelines for the interpretation of sequence variants: A joint consensus recommendation of the American College of Medical Genetics and Genomics and the Association for Molecular Pathology. Genet. Med..

[28] Baux D., Van Goethem C., Ardouin O., Guignard T., Bergougnoux A., Koenig M., Roux A.F. (2020). MobiDetails: Online DNA variants interpretation. Eur. J. Hum. Genet..

[29] VCV002854449.2-ClinVar—NCBI. https://www.ncbi.nlm.nih.gov/clinvar/variation/2854449/?oq=%22c.4357C%3EA%22[VARNAME]+%22PXDN%22[GENE]&m=NM_012293.3(PXDN):c.4357C%3EA%20(p.Pro1453Thr)%3Fterm=c.4357C%3EA%20%20PXDN.

[30] Wang R., Wiggs J.L. (2014). Common and Rare Genetic Risk Factors for Glaucoma. Cold Spring Harb. Perspect. Med..

[31] Kaushik S., Dubey S., Choudhary S., Ratna R., Pandav S.S., Khan A.O. (2022). Anterior segment dysgenesis: Insights into the genetics and pathogenesis. Indian J. Ophthalmol..

[32] Plášilová M., Stoilov I., Sarfarazi M., Kádasi L., Feráková E., Ferák V. (1999). Identification of a single ancestral CYP1B1 mutation in Slovak Gypsies (Roms) affected with primary congenital glaucoma. J. Med. Genet..

[33] Badeeb O.M., Micheal S., Koenekoop R.K., den Hollander A.I., Hedrawi M.T. (2014). CYP1B1 mutations in patients with primary congenital glaucoma from Saudi Arabia. BMC Med. Genet..

[34] Colomb E., Kaplan J., Garchon H.J. (2003). Novel cytochrome P450 1B1 (CYP1B1) mutations in patients with primary congenital glaucoma in France. Hum. Mutat..

[35] Ivanoshchuk D.E., Mikhailova S.V., Fenkova O.G., Shakhtshneider E.V., Fursova A.Z., Bychkov I.Y., Voevoda M.I. (2020). Screening of West Siberian patients with primary congenital glaucoma for CYP1B1 gene mutations. Vavilov J. Genet. Breed..

[36] Lang E., Koller S., Bähr L., Töteberg-Harms M., Atac D., Roulez F., Bahr A., Steindl K., Feil S., Berger W. (2020). Exome Sequencing in a Swiss Childhood Glaucoma Cohort Reveals CYP1B1 and FOXC1 Variants as Most Frequent Causes. Transl. Vis. Sci. Technol..

[37] Sutter T.R., Tang Y.M., Hayes C.L., Wo Y.Y., Jabs E.W., Li X., Yin H., Cody C.W., Greenlee W.F. (1994). Complete cDNA sequence of a human dioxin-inducible mRNA identifies a new gene subfamily of cytochrome P450 that maps to chromosome 2. J. Biol. Chem..

[38] Vincent A.L., Billingsley G., Buys Y., Levin A.V., Priston M., Trope G., Williams-Lyn D., Héon E. (2002). Digenic inheritance of early-onset glaucoma: CYP1B1, a potential modifier gene. Am. J. Hum. Genet..

[39] Chavarria-Soley G., Sticht H., Aklillu E., Ingelman-Sundberg M., Pasutto F., Reis A., Rautenstrauss B. (2008). Mutations in CYP1B1 cause primary congenital glaucoma by reduction of either activity or abundance of the enzyme. Hum. Mutat..

[40] Medina-Trillo C., Ferre-Fernández J.J., Aroca-Aguilar J.D., Bonet-Fernández J.M., Escribano J. (2016). Functional characterization of eight rare missense CYP1B1 variants involved in congenital glaucoma and their association with null genotypes. Acta Ophthalmol..

[41] Amirmokhtari N., Foresi B.D., Dewan S.S., Bouhenni R.A., Smith M.A. (2021). Absence of Cytochrome P450-1b1 Increases Susceptibility of Pressure-Induced Axonopathy in the Murine Retinal Projection. Front. Cell Dev. Biol..

[42] Libby R.T., Smith R.S., Savinova O.V., Zabaleta A., Martin J.E., Gonzalez F.J., John S.W. (2003). Modification of ocular defects in mouse developmental glaucoma models by tyrosinase. Science.

[43] Williams A.L., Eason J., Chawla B., Bohnsack B.L. (2017). Cyp1b1 Regulates Ocular Fissure Closure Through a Retinoic Acid-Independent Pathway. Invest. Ophthalmol. Vis. Sci..

[44] Kelberman D., Islam L., Jacques T.S., Russell-Eggitt I., Bitner-Glindzicz M., Khaw P.T., Nischal K.K., Sowden J.C. (2011). CYP1B1-related anterior segment developmental anomalies novel mutations for infantile glaucoma and von Hippel’s ulcer revisited. Ophthalmology.

[45] Tanwar M., Dada T., Dada R. (2010). Axenfeld-Rieger Syndrome Associated with Congenital Glaucoma and Cytochrome P4501B1 Gene Mutations. Case Rep. Med..

[46] Chavarria-Soley G., Michels-Rautenstrauss K., Caliebe A., Kautza M., Mardin C., Rautenstrauss B. (2006). Novel CYP1B1 and known PAX6 mutations in anterior segment dysgenesis (ASD). J. Glaucoma.

[47] Vincent A., Billingsley G., Priston M., Williams-Lyn D., Sutherland J., Glaser T., Oliver E., Walter M.A., Heathcote G., Levin A. (2001). Phenotypic heterogeneity of CYP1B1: Mutations in a patient with Peters’ anomaly. J. Med. Genet..

[48] Khan A.O., Aldahmesh M.A., Al-Abdi L., Mohamed J.Y., Hashem M., Al-Ghamdi I., Alkuraya F.S. (2011). Molecular characterization of newborn glaucoma including a distinct aniridic phenotype. Ophthalmic Genet..

[49] Melki R., Colomb E., Lefort N., Brézin A.P., Garchon H.J. (2004). CYP1B1 mutations in French patients with early-onset primary open-angle glaucoma. J. Med. Genet..

[50] Reis L.M., Tyler R.C., Weh E., Hendee K.E., Kariminejad A., Abdul-Rahman O., Ben-Omran T., Manning M.A., Yesilyurt A., McCarty C.A. (2016). Analysis of CYP1B1 in pediatric and adult glaucoma and other ocular phenotypes. Mol. Vis..

[51] Simões M.J., Carmona S., Roberts R., Wainwright G., Faro C., Silva E., Egas C. (2017). CYP1B1 mutational screening in a Portuguese cohort of primary congenital glaucoma patients. Ophthalmic Genet..

[52] Sarfarazi M., Stoilov I. (2000). Molecular genetics of primary congenital glaucoma. Eye.

[53] Kumar A., Han Y., Oatts J.T. (2024). Genetic changes and testing associated with childhood glaucoma: A systematic review. PLoS ONE.

[54] Giuffre’ I. (2011). Molecular analysis of Italian patients with congenital glaucoma. Ophthalmic Genet..

[55] Weisschuh N., Wolf C., Wissinger B., Gramer E. (2009). A clinical and molecular genetic study of German patients with primary congenital glaucoma. Am. J. Ophthalmol..

[56] Grønskov K., Redó-Riveiro A., Sandfeld L., Zibrandtsen N., Harris P., Bach-Holm D., Tümer Z. (2016). CYP1B1 Mutations in Individuals with Primary Congenital Glaucoma and Residing in Denmark. J. Glaucoma..

[57] Chouiter L., Nadifi S. (2017). Analysis of CYP1B1 Gene Mutations in Patients with Primary Congenital Glaucoma. J. Pediatr. Genet..

[58] Kushniarevich A., Utevska O., Chuhryaeva M., Agdzhoyan A., Dibirova K., Uktveryte I., Möls M., Mulahasanovic L., Pshenichnov A., Frolova S. (2015). Genetic Heritage of the Balto-Slavic Speaking Populations: A Synthesis of Autosomal, Mitochondrial and Y-Chromosomal Data. PLoS ONE.

[59] Allentoft M.E., Sikora M., Refoyo-Martínez A., Irving-Pease E.K., Fischer A., Barrie W., Ingason A., Stenderup J., Sjögren K.G., Pearson A. (2023). Population genomics of postglacial western eurasia. bioRxiv.

[60] Urnikyte A., Flores-Bello A., Mondal M., Molyte A., Comas D., Calafell F., Bosch E., Kučinskas V. (2019). Patterns of genetic structure and adaptive positive selection in the Lithuanian population from high-density SNP data. Sci. Rep..

[61] Ava S., Demirtaş A.A., Karahan M., Erdem S., Oral D., Keklikçi U. (2021). Genetic analysis of patients with primary congenital glaucoma. Int. Ophthalmol..

[62] Mears A.J., Jordan T., Mirzayans F., Dubois S., Kume T., Parlee M., Ritch R., Koop B., Kuo W.L., Collins C. (1998). Mutations of the forkhead/winged-helix gene, FKHL7, in patients with Axenfeld-Rieger anomaly. Am. J. Hum. Genet..

[63] Saleem R.A., Banerjee-Basu S., Berry F.B., Baxevanis A.D., Walter M.A. (2003). Structural and functional analyses of disease-causing missense mutations in the forkhead domain of FOXC1. Hum. Mol. Genet..

[64] Nishimura D.Y., Swiderski R.E., Alward W.L., Searby C.C., Patil S.R., Bennet S.R., Kanis A.B., Gastier J.M., Stone E.M., Sheffield V.C. (1998). The forkhead transcription factor gene FKHL7 is responsible for glaucoma phenotypes which map to 6p25. Nat. Genet..

[65] Valleix S., Niel F., Nedelec B., Algros M.P., Schwartz C., Delbosc B., Delpech M., Kantelip B. (2006). Homozygous nonsense mutation in the FOXE3 gene as a cause of congenital primary aphakia in humans. Am. J. Hum. Genet..

[66] Doucette L., Green J., Fernandez B., Johnson G.J., Parfrey P., Young T.L. (2011). A novel, non-stop mutation in FOXE3 causes an autosomal dominant form of variable anterior segment dysgenesis including Peters anomaly. Eur. J. Hum. Genet..

[67] Khan K., Rudkin A., Parry D.A., Burdon K.P., McKibbin M., Logan C.V., Abdelhamed Z.I., Muecke J.S., Fernandez-Fuentes N., Laurie K.J. (2011). Homozygous mutations in PXDN cause congenital cataract, corneal opacity, and developmental glaucoma. Am. J. Hum. Genet..

[68] Khan K., Al-Maskari A., McKibbin M., Carr I.M., Booth A., Mohamed M., Siddiqui S., Poulter J.A., Parry D.A., Logan C.V. (2011). Genetic heterogeneity for recessively inherited congenital cataract microcornea with corneal opacity. Invest. Ophthalmol. Vis. Sci..

[69] Choi A., Lao R., Ling-Fung Tang P., Wan E., Mayer W., Bardakjian T., Shaw G.M., Kwok P.Y., Schneider A., Slavotinek A. (2015). Novel mutations in PXDN cause microphthalmia and anterior segment dysgenesis. Eur. J. Hum. Genet..

[70] Micheal S., Siddiqui S.N., Zafar S.N., Iqbal A., Khan M.I., Den Hollander A.I. (2016). Identification of Novel Variants in LTBP2 and PXDN Using Whole-Exome Sequencing in Developmental and Congenital Glaucoma. PLoS ONE.

[71] Xia K., Wu L., Liu X., Xi X., Liang D., Zheng D., Cai F., Pan Q., Long Z., Dai H. (2004). Mutation in PITX2 is associated with ring dermoid of the cornea. J. Med. Genet..

[72] Doward W., Perveen R., Lloyd I.C., Ridgway A.E.A., Wilson L., Black G.C.M. (1999). A mutation in the RIEG1 gene associated with Peters’ anomaly. J. Med. Genet..

[73] Alward W.L., Semina E.V., Kalenak J.W., Héon E., Sheth B.P., Stone E.M., Murray J.C. (1998). Autosomal dominant iris hypoplasia is caused by a mutation in the Rieger syndrome (RIEG/PITX2) gene. Am. J. Ophthalmol..

[74] Bidinost C., Matsumoto M., Chung D., Salem N., Zhang K., Stockton D.W., Khoury A., Megarbane A., Bejjani B.A., Traboulsi E.I. (2006). Heterozygous and homozygous mutations in PITX3 in a large Lebanese family with posterior polar cataracts and neurodevelopmental abnormalities. Investig. Ophthalmol. Vis. Sci..

[75] Semina E.V., Ferrell R.E., Mintz-Hittner H.A., Bitoun P., Alward W.L.M., Reiter R.S., Funkhauser C., Daack-Hirsch S., Murray J.C. (1998). A novel homeobox gene PITX3 is mutated in families with autosomal-dominant cataracts and ASMD. Nat. Genet..

[76] Deml B., Reis L.M., Lemyre E., Clark R.D., Kariminejad A., Semina E.V. (2016). Novel mutations in PAX6, OTX2 and NDP in anophthalmia, microphthalmia and coloboma. Eur. J. Hum. Genet..

[77] Mirzayans F., Pearce W.G., MacDonald I.M., Walter M.A. (1995). Mutation of the PAX6 gene in patients with autosomal dominant keratitis. Am. J. Hum. Genet..

[78] Jordan T., Hanson I., Zaletayev D., Hodgson S., Prosser J., Seawright A., Hastie N., van Heyningen V. (1992). The human PAX6 gene is mutated in two patients with aniridia. Nat. Genet..

[79] Bonet-Fernández J.M., Aroca-Aguilar J.D., Corton M., Ramírez A.I., Alexandre-Moreno S., García-Antón M.T., Salazar J.J., Ferre-Fernández J.J., Atienzar-Aroca R., Villaverde C. (2020). CPAMD8 loss-of-function underlies non-dominant congenital glaucoma with variable anterior segment dysgenesis and abnormal extracellular matrix. Hum. Genet..

[80] Cheong S.S., Hentschel L., Davidson A.E., Gerrelli D., Davie R., Rizzo R., Pontikos N., Plagnol V., Moore A.T., Sowden J.C. (2016). Mutations in CPAMD8 Cause a Unique Form of Autosomal-Recessive Anterior Segment Dysgenesis. Am. J. Hum. Genet..

[81] Kopanos C., Tsiolkas V., Kouris A., Chapple C.E., Albarca Aguilera M., Meyer R., Massouras A. (2019). VarSome: The human genomic variant search engine. Bioinformatics.

[82] Siggs O.M., Souzeau E., Pasutto F., Dubowsky A., Smith J.E., Taranath D., Pater J., Rait J.L., Narita A., Mauri L. (2019). Prevalence of FOXC1 Variants in Individuals with a Suspected Diagnosis of Primary Congenital Glaucoma. JAMA Ophthalmol..

[83] Chakrabarti S., Ghanekar Y., Kaur K., Kaur I., Mandal A.K., Rao K.N., Parikh R.S., Thomas R., Majumder P.P. (2010). A polymorphism in the CYP1B1 promoter is functionally associated with primary congenital glaucoma. Hum. Mol. Genet..

